# A case report in hypertrophic cardiomyopathy in a recreational athlete: multimodality risk assessment, genetic insights, and shared decision-making

**DOI:** 10.1093/ehjcr/ytaf671

**Published:** 2025-12-23

**Authors:** Luis Enrique Gomez, Eliana Aversa, Maria Mercedes Saenz Tejeira, Paula Buonfiglio, Nicolas Atamañuk

**Affiliations:** Hospital de Agudos Juan A. Fernández, Av. Cerviño 3356, CP: 1425AGP, Ciudad Autonoma de Buenos Aires, Argentina; Hospital de Agudos Juan A. Fernández, Av. Cerviño 3356, CP: 1425AGP, Ciudad Autonoma de Buenos Aires, Argentina; Hospital de Agudos Juan A. Fernández, Av. Cerviño 3356, CP: 1425AGP, Ciudad Autonoma de Buenos Aires, Argentina; Hospital de Agudos Juan A. Fernández, Av. Cerviño 3356, CP: 1425AGP, Ciudad Autonoma de Buenos Aires, Argentina; Hospital de Agudos Juan A. Fernández, Av. Cerviño 3356, CP: 1425AGP, Ciudad Autonoma de Buenos Aires, Argentina

**Keywords:** Hypertrophic cardiomyopathy, Athlete, Sports cardiology, Genetic testing, Risk stratification, ICD, MYBPC3, Case report

## Abstract

**Background:**

Sports participation in patients with inherited cardiac conditions remains a major challenge, particularly when balancing arrhythmic risk against quality of life.

**Case summary:**

A case of a 38-year-old recreational athlete diagnosed with hypertrophic cardiomyopathy (HCM) is described. A comprehensive assessment was performed, including electrocardiogram, echocardiography, exercise stress testing, Holter monitoring, and cardiac magnetic resonance, which revealed asymmetric non-obstructive HCM with extensive late gadolinium enhancement. Genetic testing identified two missense variants in *MYBPC3*, one of which was reclassified as *likely pathogenic*. Risk stratification using European Society of Cardiology (ESC) and American Heart Association/American College of Cardiology (AHA/ACC) calculators demonstrated a 5.73% 5-year risk of sudden cardiac death (SCD). A subcutaneous implantable cardioverter-defibrillator was implanted after a shared decision-making process, allowing the patient to continue recreational sport while avoiding competitive athletics.

**Discussion:**

This case highlights the value of multimodal imaging, genetic testing, and guideline-based SCD risk stratification in guiding individualized management of athletes with HCM.

Learning points This case therefore illustrates five teaching points:

- Multimodal imaging, particularly cardiac magnetic resonance, is critical in identifying fibrosis and refining risk in HCM.
- Holter monitoring provides evidence of arrhythmia and contributes to sudden cardiac death risk stratification.
- Genetic testing can uncover variants in critical domains of *MYBPC3*, supporting personalized care.
- Subcutaneous implantable cardioverter-defibrillators are valuable in balancing arrhythmic protection with quality of life in active patients.
- Shared decision-making remains fundamental in managing athletes with inherited cardiomyopathies.

## Introduction

Hypertrophic cardiomyopathy (HCM) is a hereditary heart disease characterized by left ventricular hypertrophy (≥15 mm) in the absence of abnormal loading conditions.^1,2^ It affects approximately 1 in 500 individuals^3^ and follows an autosomal dominant inheritance pattern, most commonly involving variants in *MYH7* and *MYBPC3* genes.^4^ Echocardiography remains the cornerstone of diagnosis, while cardiac magnetic resonance (CMR) provides incremental prognostic value by detecting late gadolinium enhancement, apical aneurysms, and microvascular dysfunction. Sudden cardiac death (SCD) risk stratification relies on validated models such as the HCM Risk-SCD^5^ calculators to guide implantable cardioverter-defibrillator (ICD) therapy. Advances in imaging, genetics, and novel targeted therapies have contributed to a decline in annual mortality to around 0.5%,^6^ underscoring the evolution of HCM into a largely manageable condition with expanding opportunities for precision medicine.

Inherited cardiac conditions (ICCs), particularly HCM, represents a major cause of SCD in athletes. Early detection remains difficult, highlighting the importance of multimodal evaluation. Genetic testing and multidisciplinary input now play a key role in refined risk stratification and tailored recommendations on sports participation.

In this work, we aimed to describe the comprehensive multimodal evaluation and personalized management of a young athlete with HCM carrying two *MYBPC3* variants, extensive myocardial fibrosis, and a moderate risk of SCD managed with subcutaneous ICD implantation.

## Summary figure

**Figure:**
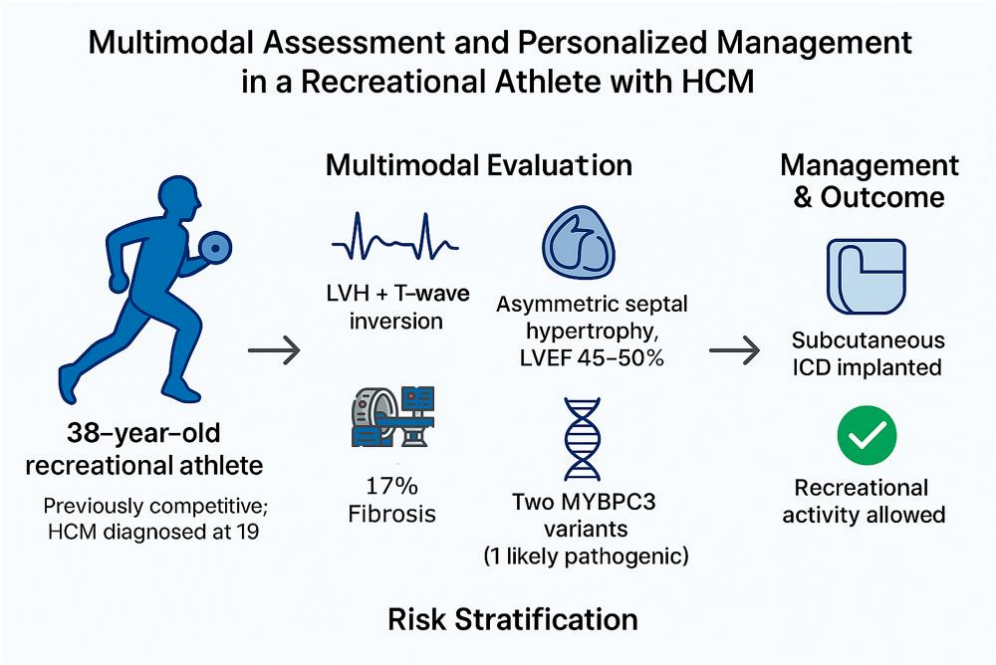


## Case presentation

A 38-year-old man, previously a competitive athlete and, as of 2021, engaged in recreational sports, was referred to our institution for re-evaluation of HCM, initially diagnosed during a pre-participation screening in 2006. There was no family history of cardiomyopathy or SCD. He starts to follow up in our centre in April 2021. At the time of consultation, the patient was a weightlifter with 15 years of training experience and followed a structured resistance training regimen (approximately five sessions per week).

The patient was specifically questioned about the use of dietary supplements and denied taking any ergogenic or anabolic agents, including anabolic-androgenic steroids (AAS), testosterone derivatives, or other hormonal products. At physical evaluation, there were no clinical signs suggestive of AAS use, such as acne, striae distensae, gynaecomastia, testicular atrophy, or hypertension. Laboratory findings, including HDL cholesterol, haematocrit, and γ-glutamyl transpeptidase, were within normal limits, without evidence of erythrocytosis, consistent with the absence of indirect markers of AAS abuse in athletes^7^

On physical examination, the patient was asymptomatic. Blood pressure was 130/60 mmHg, heart rate 67 b.p.m., and oxygen saturation 99%. No murmurs were present at rest or with Valsalva, and there were no signs of heart failure.

**Figure 1:**
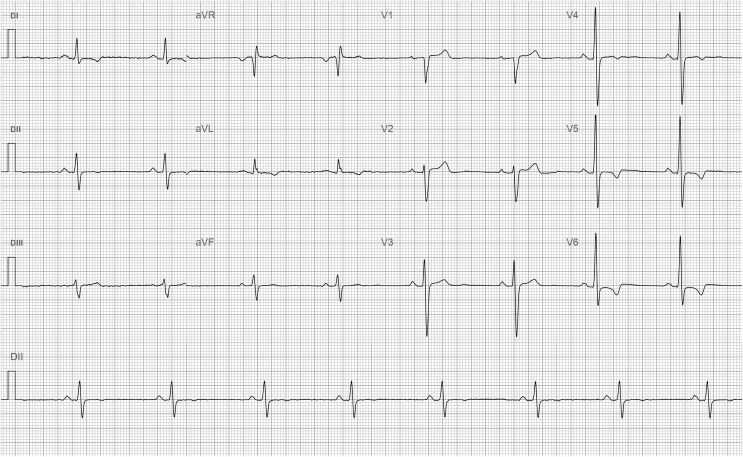
Resting electrocardiogram showing sinus rhythm with left ventricular hypertrophy (Romhilt–Estes criteria) and T-wave inversion inferolateral leads (I, II, aVL, V4–V6).

The resting electrocardiogram demonstrated sinus rhythm with features typical of HCM, including score criteria for left ventricular hypertrophy and repolarization abnormalities with T-wave inversion in the inferolateral leads (I, II, aVL, V4–V6) (*Figure 1*).

Transthoracic echocardiography revealed normal left ventricular diameters (LVEDD 47.7 mm LVESD 24.5 mm) with asymmetric septal hypertrophy (24 mm), mild left ventricular ejection fraction (LVEF 45%–50%), and biatrial enlargement. Stress echocardiography showed no ischaemia or dynamic left ventricular outflow tract obstruction.

**Figure 2:**
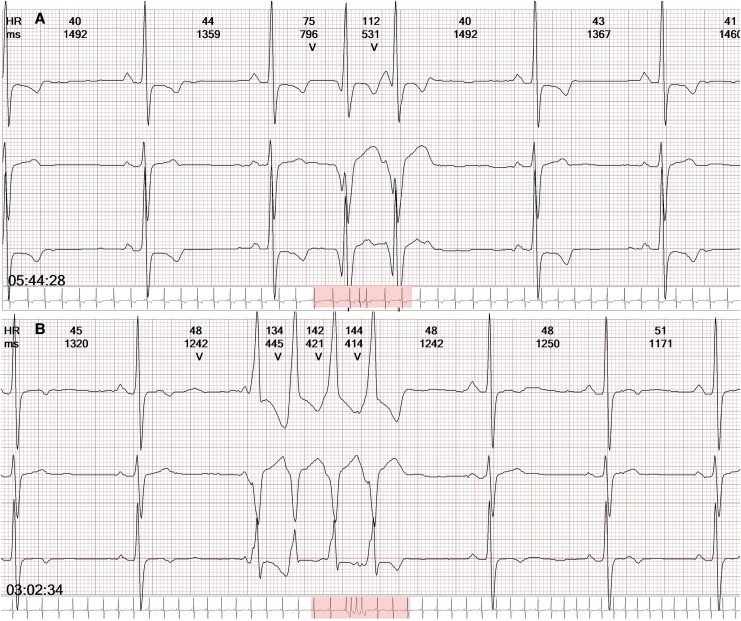
24-h Holter monitoring demonstrating ventricular couplets (*A*) and non-sustained ventricular tachycardia (*B*) (four beats).

A 24-h Holter monitoring revealed sinus rhythm with an average heart rate of 57 b.p.m., 230 monomorphic premature ventricular contractions, and one short run of non-sustained ventricular tachycardia lasting four beats (*Figure 2*).

**Figure 3:**
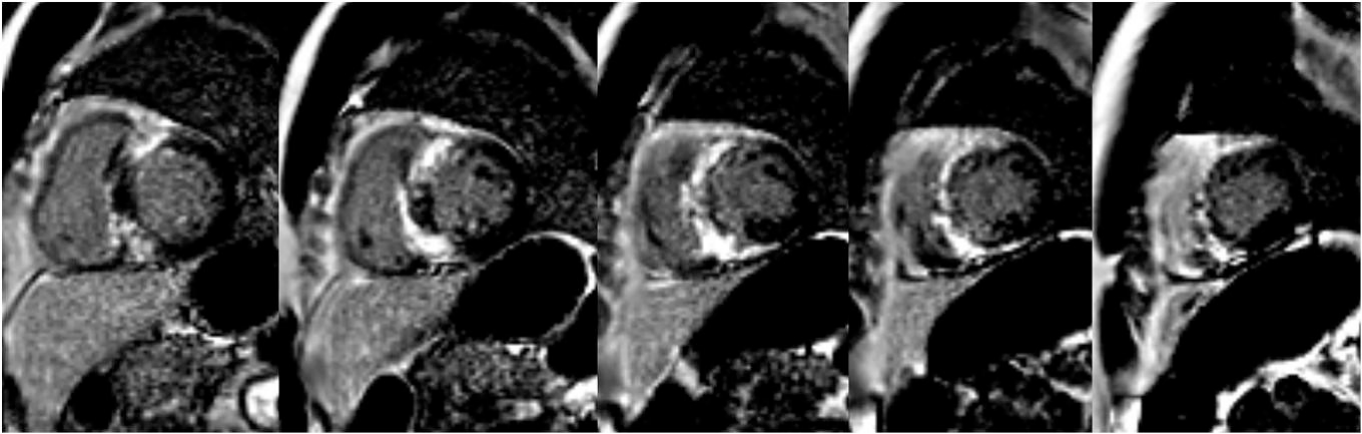
Cardiac magnetic resonance showing late gadolinium enhancement in the basal septal and mid-septal and anterior segments, involving 17% of the myocardial mass.

To refine phenotypic characterization, a CMR imaging was performed. Cardiac magnetic resonance confirmed preserved biventricular systolic function (LVEF 59%) and asymmetric septal hypertrophy with a maximum thickness of 19 mm. In addition, marked biatrial enlargement was evident, with a left atrial volume index of 79 mL/m² and right atrial area of 25 cm². Importantly, extensive diffuse late gadolinium enhancement (LGE) was detected in the basal and mid-septal and anterior segments, involving 17% of the myocardial mass (*Figure 3*).

To complement imaging findings, genetic testing was performed using the Health in Code HCM panel, which includes the following 18 genes: *ACTC1*, *DES*, *FHL1*, *FHOD3*, *GLA*, *LAMP2*, *MYBPC3*, *MYH7*, *MYL2*, *MYL3*, *PRKAG2*, *PTPN11*, *TNNC1*, *TNNI3*, *TNNT2*, *TPM1*, *TRIM63*, and *TTR*. Two missense variants in *MYBPC3* (NM_000256.3): c.2533C>G in exon 26 (C6 domain) were identified and, classified as a variant of uncertain significance (VUS, score 2), and c.1696T>A in exon 18 (C4 domain), a VUS with a score of 5.

Finally, risk stratification with the ESC HCM Risk-SCD calculator estimated a 5-year SCD risk of 5.73%. Upon meeting the threshold for device implantation, we decided in the heart team, after a shared decision-making process with the patient, to proceed with the placement of a subcutaneous ICD. He was advised to avoid competitive or high-intensity sports, while recreational activity was permitted. At follow-up, the patient has remained clinically stable and free of arrhythmic events with the echocardiogram still showing a LVEF of 50%.

## Discussion

This case illustrates the complexities of managing athletes with inherited cardiomyopathies. Despite being asymptomatic, the patient exhibited several markers of risk: non-sustained ventricular tachycardia, biatrial enlargement, extensive fibrosis on CMR, and two *MYBPC3* variants located in critical structural regions (C4 and C6 domains). Variants in these domains are known to impair cardiac myosin-binding protein C (cMyBP-C) function, potentially disrupting sarcomeric contraction.^8^ The coexistence of both variants may have contributed to the patient’s aggressive phenotype, characterized by extensive fibrosis and NSVT. While one of the variants lacks sufficient evidence to be considered clinically significant (VUS, score 2), the variant c.1696T>A scored 5 points, just one point below the threshold for classification as likely pathogenic. Recently, it has been proposed that such variants be classified as HOT-VUS, since this type of variant is likely to be reclassified towards pathogenicity as additional evidence becomes available.^9^ This variant should therefore be periodically re-evaluated, as the current evidence provides further support for its potential role in the patient’s phenotype.

Multimodal imaging was central to risk stratification, with CMR quantifying myocardial fibrosis as an independent prognostic marker. Cardiac magnetic resonance is considered the gold-standard non-invasive modality for myocardial tissue characterization, including fibrosis.^10^ The discrepancy in LVEF values between echocardiography and CMR is likely attributable to the technical limitations of echocardiographic assessment in markedly hypertrophied ventricles. In this context, accurate evaluation of systolic function can be particularly challenging, with substantial operator-dependent variability. Therefore, CMR is routinely performed in most HCM patients at our institution to ensure precise volumetric and functional assessment. Notably, the burden exceeded the 15% threshold, a level consistently associated with heightened arrhythmic risk.^11^ Holter monitoring documented arrhythmia, adding further weight to the risk profile.^12^ In our patient, the ESC HCM Risk-SCD calculator estimated a 5-year risk of SCD of 5.73%, which corresponds to a Class IIb recommendation for ICD implantation according to the 2023 ESC Guidelines for the management of cardiomyopathies. Additionally, the presence of extensive LGE (>15%) also represents a Class IIb indication for ICD.^13^ Although the HCM Risk-SCD calculator has not been validated in athletic populations, there is no evidence that athletes with HCM have a lower arrhythmic risk; indeed, exercise-related physiological stress may confer equal or greater susceptibility to malignant ventricular arrhythmias. Therefore, the relevance of these markers of risk is not diminished in this setting and may even reinforce the rationale for ICD implantation. Consequently, device implantation was guided by a comprehensive multidisciplinary assessment integrating imaging, clinical evaluation, genetic testing, and shared decision-making.^14^ In addition to this, genetic testing not only provided diagnostic certainty but also revealed variant clustering in genomic regions associated with adverse phenotypes. Furthermore, the presence of double variants highlighted the potential for compounded effects on disease severity, underscoring the importance of comprehensive sequencing approaches for accurate risk stratification.^15,16^

Integration of these findings allowed personalized management. The implantation of a subcutaneous ICD balanced sudden death prevention with the patient’s wish to remain physically active. Regarding athletic activity, competitive or high-intensity sports were discouraged due to the elevated arrhythmic risk, while recreational activity was permitted under supervision. The implantation of a subcutaneous ICD was chosen to provide arrhythmic protection while minimizing long-term risks associated with transvenous leads, particularly in a young, physically active patient. The final decision integrated multiple risk factors: arrhythmia burden, fibrosis extent, genetic findings, and calculated SCD risk, highlighting the necessity of an individualized, multidisciplinary approach in athletes with HCM. This case aligns with current ESC and AHA/ACC guidelines, which encourage individualized recommendations and shared decision-making rather than categorical restrictions for patients with ICCs.

## Conclusion

Athletes with HCM require a comprehensive, multimodal evaluation that integrates imaging, arrhythmia monitoring, and genetic testing. The identification of multiple variants in *MYBPC3*, combined with imaging markers of fibrosis, refined the risk stratification and supported the indication for ICD implantation. Shared decision-making was essential to establish a management plan that ensured arrhythmic protection while permitting safe participation in recreational activity.

## Lead author biography


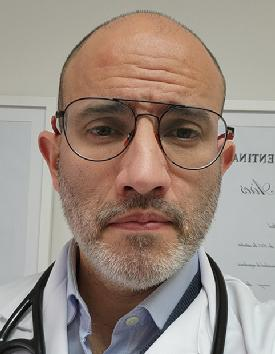


I earned my medical degree from the University of Buenos Aires in 2005 and completed cardiology training at the Italian Hospital of Buenos Aires, obtaining a post-graduate degree in 2010. Since 2012, I have served at Hospital de Agudos ‘Juan A. Fernández’, where I specialized in congenital heart disease, later earning my paediatric cardiology title (2016). In 2020, I pursued a master’s in genetics and founded the Hereditary Cardiovascular Diseases Clinic. Currently, I lead adult congenital programmes at two major hospitals, serve as Scientific Secretary of the Genetic Cardiology Council, teach, mentor, and actively contribute to research and academia.

## Data Availability

Data sharing is not applicable to this article as no new data were created or analysed in this study.
